# Peripheral Cytotoxic T Cell Lymphoma of the Appendix Presenting as Acute Appendicitis

**DOI:** 10.1155/2020/8569426

**Published:** 2020-06-16

**Authors:** Negin Rahmani, Yahya Daneshbod, Hadi Mirfazaelian, Elnaz Vahidi, Sadegh Shirian

**Affiliations:** ^1^Shiraz University of Medical Sciences, Shiraz, Iran; ^2^Shiraz Molecular Pathology Research Center, Dr. Daneshbod Lab, Shiraz, Iran; ^3^Department of Pathology and Laboratory Medicine, Loma Linda University, California, USA; ^4^Emergency Medicine Department, Tehran University of Medical Sciences, Tehran, Iran; ^5^Department of Pathology, School of Veterinary Medicine, Shahrekord University, Shahrekord, Iran; ^6^Biotechnology Research Institute, Shahrekord University, Shahrekord, Iran; ^7^Shefa Neuroscience Research Center, Tehran, Iran

## Abstract

**Introduction:**

Lymphoma of the appendix is a rare cause of acute appendicitis; however, acute appendicitis is a common first manifestation of appendiceal lymphomas. Cytotoxic peripheral T cell lymphoma (PTCL) is a type of aggressive non-Hodgkin lymphoma that portends a generally poor outcome. Cytotoxic PTCL of the appendix is extremely rare with few cases reported in the literature. *Case Presentation*. This is the report of a 23-year-old man who had experienced lower abdominal pain for three months before presenting to the emergency department with severe right lower abdominal pain, nausea, vomiting, and anorexia since the day prior to admission. The patient was diagnosed with acute appendicitis, and the pathology report confirmed cytotoxic PTCL of the appendix.

**Conclusion:**

Patients with appendiceal PTCL commonly present with signs and symptoms of acute appendicitis due to luminal obstruction by the tumor. Therefore, appendiceal tumors such as PTCL should be considered in the differential diagnosis of patients presenting as acute appendicitis. In addition, since there is no standard chemotherapy regimen for cytotoxic PTCL, this and other case reports hopefully help in providing the clinical evidence needed for establishing appropriate treatment guidelines.

## 1. Background

Acute appendicitis is one of the most common causes of acute abdomen worldwide [1]. One rare cause of acute appendicitis is the lymphoma of the appendix. Although appendiceal lymphoma is itself a rare phenomenon [2–4], acute appendicitis is a very common presentation in this and other tumors of the appendix [3, 4]. Peripheral T cell lymphomas (PTCLs) are a group of heterogeneous lymphoproliferative disorders that originate from peripheral T cells or mature Natural Killer (NK) cells and are a subgroup of non-Hodgkin lymphomas (NHLs) [5]. PTCLs of cytotoxic T cell origin are rare entities, and appendiceal cytotoxic PTCL is even rarer. Here, we introduce a case of appendiceal cytotoxic PTCL presenting as acute appendicitis.

## 2. Case Presentation

A 23-year-old man came to the emergency department because of right lower quadrant (RLQ) abdominal pain. He had a history of 3 months of vague lower abdominal pain, which became progressive and was more severe since the day prior to admission. It was associated with anorexia, nausea, and vomiting. He had no urinary or bowel changes and he had no significant past medical history. He reported that his brother had died at 46 years of age, but the reason was not determined.

On physical examination, he was ill with generalized malaise. He had a pulse rate of 105, blood pressure of 110/55 mmHg, respiratory rate of 18, and oral temperature 38.1°C. RLQ abdominal tenderness and guarding were present. The physical examination was otherwise insignificant.

The patient was admitted to the surgery ward with the impression of acute appendicitis. Appendectomy was performed and acute appendicitis was confirmed. However, the patient's condition started to deteriorate and further workup was performed. Ultrasound examination of the abdomen and pelvis showed multiple pathologic lymph nodes with varying sizes at the right lower quadrant with the largest one measuring 20 mm. In addition, mild free fluid between bowel loops was observed.

Abdominopelvic CT scan revealed thickened small and large intestinal wall as well as lymphadenopathy. The pathology sample was reviewed and sent for further molecular analysis which revealed the final diagnosis, i.e., PTCL of the appendix. Unfortunately, the patient died four weeks after admission during the first round of chemotherapy.

## 3. Pathology, Immunohistochemistry, and Genetic Analysis

**Figure 1:**
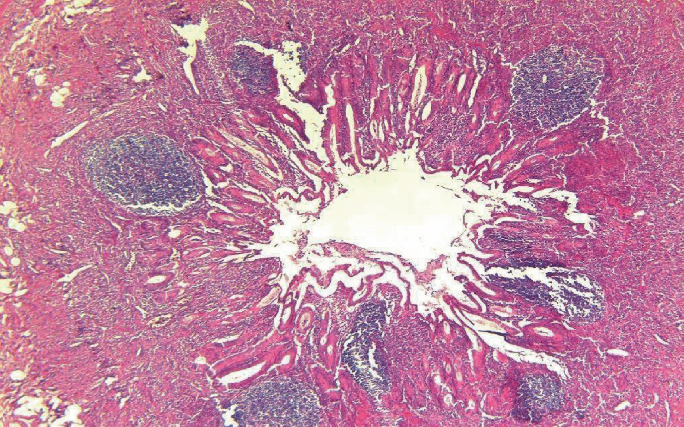
Appendiceal mucosa diffusely infiltrated by atypical cells. The muscular layer shows neutrophilic infiltration. Hematoxylin and eosin, ×4.

**Figure 2:**
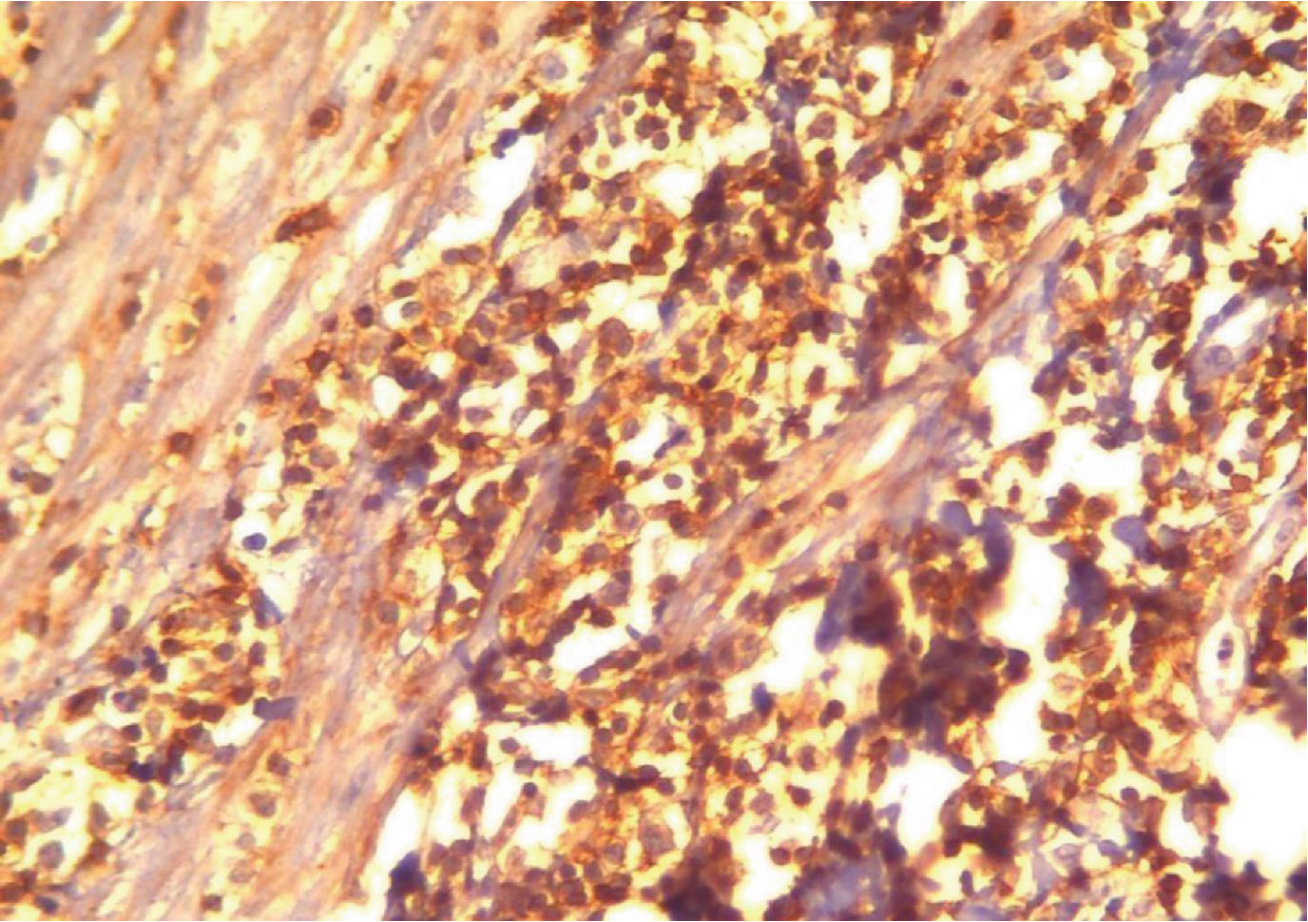
Atypical lymphoid cells are positive for CD3. Immunoperoxidase stain.

**Figure 3:**
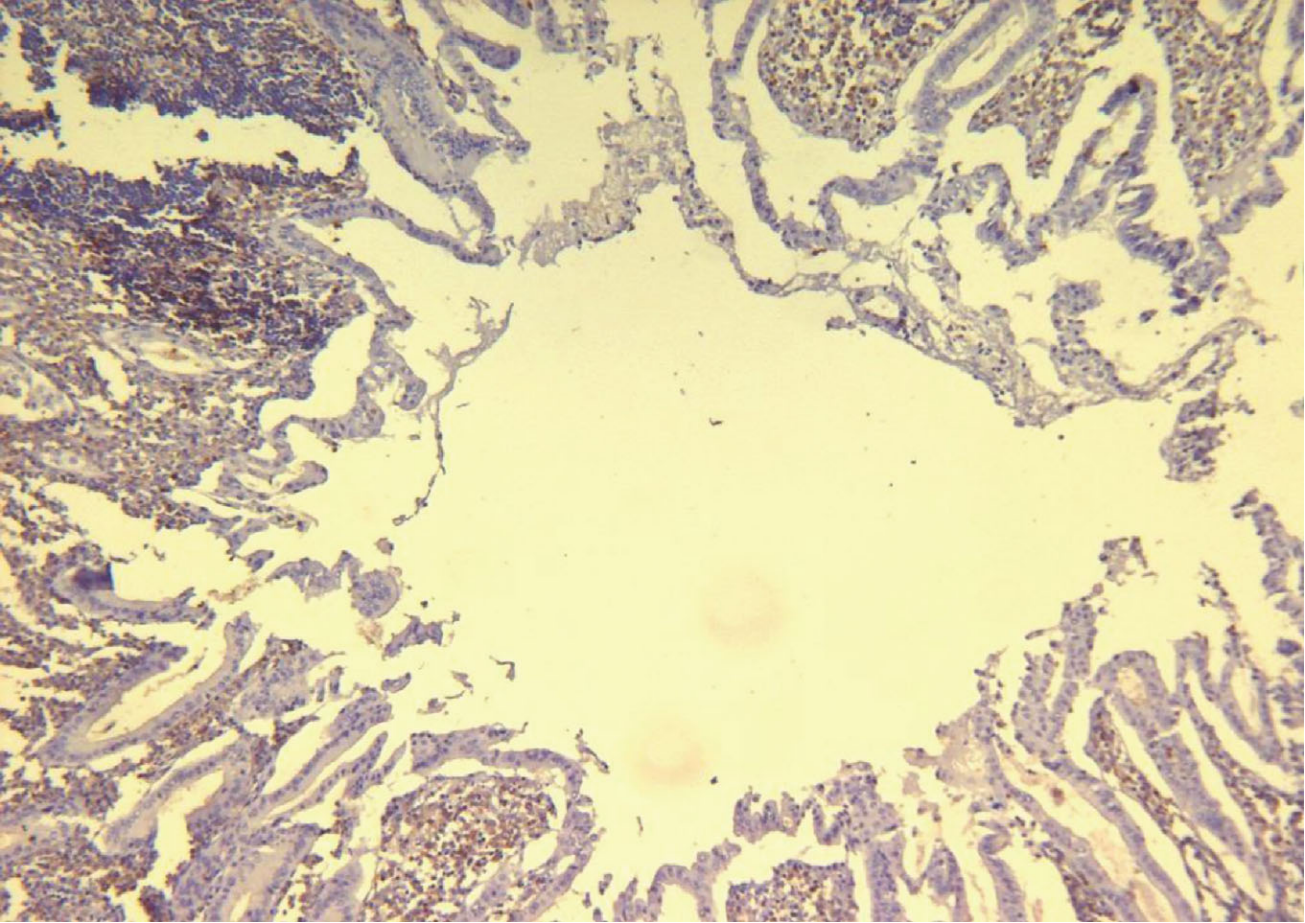
Atypical lymphoid cells are positive for CD8. Immunoperoxidase stain.

Histopathology of the appendix showed the presence of atypical lymphoid cells around mucosal lymphoid follicles in appendiceal as well as the adjacent bowel wall and also neutrophilic infiltration in the muscular layer of the wall consistent with appendicitis and periappendicitis (Figures 1–3). Diffuse thickening of the small and large intestines was also observed. Immunohistochemical analysis revealed that the atypical cells were CD3, CD2, CD7, CD8, and CD25 positive with partial loss of CD5. The cells expressed cytotoxic granules (TIA1 and Granzyme B) and were TCR *β*F1 positive. Some of the cells expressed CD30. Markers such as CD79*α*, CD20, ALK1, CD56, and FOXP3 as well as Epstein Barr Virus (EBV) “in situ” hybridization (EBER) and HHV8 were negative. The proliferative index evaluated with ki67 was high, more than 80% of the atypical cells. According to these findings, the diagnosis of cytotoxic PTCL was made.

## 4. Discussion

Appendiceal lymphoma is a rare malignancy that can present as acute appendicitis [3]. The gastrointestinal (GI) tract is considered the most common extranodal site for lymphomas [3, 6], and appendiceal lymphomas constitute about 0.015% of all gastrointestinal lymphomas [3]. The majority of appendiceal lymphomas are NHLs with B-cell NHLs being more common than NHLs with a T cell origin [7, 8]. In a study evaluating over 291,000 GI tract neoplasms, 3,597 cases of NHL were detected; thirty-six were located in the appendix with the majority being diffuse large B cell lymphoma [7]. PTCLs are a subdivision of T cell NHLs that include several entities such as anaplastic large cell lymphoma, nodal peripheral T cell lymphoma with TFH phenotype, and peripheral T cell lymphoma not otherwise specified (NOS). Compared with B cell lymphomas, they usually portend a poorer prognosis and have a higher incidence of diffuse and extranodal disease [9].

With regard to the presentation, patients with appendiceal PTCL, like other neoplasms of the appendix, commonly present with signs and symptoms of acute appendicitis due to luminal obstruction by the tumor [2–4, 10]. However, they may have atypical presentations. For example, they may have RLQ pain, lower abdominal pain, or a palpable mass in the RLQ of the abdomen for a few months [3, 11], as in our patient who had lower abdominal pain since 3 months prior to admission. PTCL patients commonly experience B symptoms (as opposed to B cell lymphoma patients), and their median age of presentation is between the 6^th^ and 7^th^ decades of life [5].

The diagnosis of appendiceal PTCL is made with imaging modalities—the most useful of which is computed tomography (CT) imaging, histopathology, immunophenotyping, and genetic analysis. CT scan in appendiceal lymphoma patients demonstrates appendiceal enlargement with the maintenance of its vermiform appearance. Diffuse mural thickening and aneurysmal dilatation of the appendiceal lumen can also be present. It is suggested that the appendix diameter exceeding 15 mm on CT scan should raise suspicion about appendiceal tumor since the diameter is less when the appendicitis is not associated with a tumor [12]. NHLs tend to infiltrate the appendix wall circumferentially and can obliterate the lumen [4]. Histological examination is a first step that is followed by immunohistochemistry, flow cytometry, cytogenetics, and molecular genetics. Polymerase Chain Reaction (PCR) for T cell receptor gene rearrangements should be performed to assess the clonality [9].

Due to the very small number of appendiceal PTCL cases, it is difficult to establish standard management guidelines for them. PTCLs, unlike B cell lymphomas, do not respond well to conventional chemotherapy and have a generally poorer outcome. The reason for this might be the fact that PTCLs disseminate early in the course of disease and that there are virtually no specific chemotherapy drugs for them and the current regimens are according to guidelines for diffuse large B cell lymphoma treatment. Treatment modalities include surgical exploration and resection, chemotherapy, and radiotherapy. The first-line chemotherapy regimen for PTCL has conventionally been Cyclophosphamide, Doxorubicin, Vincristine, and Prednisone (CHOP) or CHOP-like regimens. This regimen leads to a 5-year overall survival of 32%. Novel drugs that target specific cellular pathways have been suggested that when used in combination with the conventional chemotherapy have shown promise [9]. PTCL is a rare malignancy, and since the immunohistochemical and genetic analyses have been performed in only a few of the reported cases, the exact incidence and features of this type of lymphoma are unknown. Kitamura et al. have reported the first case of cytotoxic T cell NHL of the appendix presenting in an 86-year-old woman as acute appendicitis [8]. Matsushita and Takeshita have reported another case of cytotoxic T cell NHL of the appendix in a 7-year-old boy who had come to the hospital due to abdominal discomfort and high fever [13]. Another case was reported by Ratuapli et al. [14] in which EBV-positive T cell lymphoma was diagnosed in a renal transplant patient manifesting as acute appendicitis. This patient was initially treated with cisplatin, etoposide, solumedrol, and gemcitabine, and after a rapid response to this regimen, the chemotherapy was changed to CHOP regimen [14]. In order to understand the clinical course and prognosis of appendiceal T cell lymphoma, more cases should be identified and followed up. We emphasize having a high degree of suspicion when a patient presents with signs and symptoms of acute appendicitis but has atypical presentations, e.g., a longer than expected time course for the abdominal pain, recent weight loss, etc.

In addition, in cases where imaging such as ultrasound is performed, attention must be paid to atypical findings such as lymphadenopathy in the pelvic cavity.

## 5. Conclusion

Considering the fact that most appendiceal lymphomas initially present as acute appendicitis, physicians should always consider this disease in the differential diagnosis of acute abdomen. This is especially important since early diagnosis can change the management and surgical approach and since a late diagnosis is associated with a poorer prognosis. In addition, all appendectomy specimens in cases of acute appendicitis should be evaluated by histopathology even if they appear normal by gross examination.
